# On the Therapeutical Properties of Arsenic

**Published:** 1831

**Authors:** 


### LVII.

### ON THE THERAPEUTICAL PROPERTIES OF ARSENIC. By J. ANDERSON, Esq.

IN the second Number of the Glasgow Medical Examiner, Mr. Anderson has published some observations on arsenic, which coincide exactly with our own experience. These observations may tend to lessen the timidity with which this powerful, but safe medicine is administered by many practitioners in this country; and, as the paper is short, we shall give it in the words of the author.

"In calling the attention of my fellow-practitioners to arsenic, as a remedy, it is not my intention to give either a systematic account of its effects on the constitution, or of the diseases in which it has been employed; but to point out some peculiarities in its action, which I think will justify a more extended exhibition of this medicine, than has been usual in this part of the world.

The first point to which I would direct the attention of the practitioner, who has not been in the habit of prescribing the oxide of arsenic, is, that its action in a dose, which is poisonous, is altogether different, not only in degree, but in kind, from the effects which it produces when properly administered. Its earliest poisonous effects are, diminished action of the heart and intense inflammation of the stomach; and occasionally the latter is so severe and immediate, as to destroy life ere it has effected any of those disorganizations by which it is recognized.* Given medicinally, its effects are totally different from those it displays as a poison. The first and earliest sign of its action, is an increased strength and frequency of the pulse; next follow, the usual fulness of the palpebræ, and itching of the *alæ nasi.* If the exhibition of the medicine be continued, the irritation of the mucous membrane extends to the fauces, inducing redness and cough; and along with these symptoms, the tongue begins to be covered with a white fur, which, gradually increasing in thickness, gives its surface the appearance of having been rubbed over with chalk. The action of the heart continues to increase in force and frequency; the pulse becomes full and hard; and at last, a general anasarcous state begins to be established. Up to this point, the medicine may generally be given with safety.

The great peculiarity, then, in the action of arsenic, is the powerful effect it produces upon the heart. It is, in fact, a permanent stimulant to the vascular system, and in this consists its value as a therapeutical agent. In arsenic, the practitioner has a powerful stimulant, without any subsequent debility, the muscular force and general strength of the patient being rather increased than diminished during its use; he has it in his power to increase the fulness and strength of the pulse; to induce, in fact, a state of general plethora. Surely, a medicine possessed of properties so valuable, ought not to be looked upon with that distrust with which it is commonly regarded, and be prescribed in rare and isolated cases, with a trembling hesitation, very dif-

---

* "This explanation of the manner in which arsenic produces death, is certainly more in accordance with the known action of metallic poisons, than that it should, as Brodie and others have asserted, prove fatal by a supposed sedative action upon the heart and brain."

ferent from a well-regulated confidence in its virtues; and, farther, be abandoned, so soon as it begins to produce its necessary and specific effects upon the system. What would be the amount of our knowledge of the effects of mercury, if we always gave up its use so soon as it produced tenderness of the gums, and before ptyalism came on? About as much, or more, than we know of the effects of the oxide of arsenic.

If we institute a comparison betwixt the general effects of arsenic, as a remedy, and those of mercury, its inconveniences will be found greatly less. It causes no debility; no salivation; there is no danger from exposure to cold during its use; opium is not required to correct its action on the bowels, and the patient may, if otherwise able, pursue his usual avocations: a point of very considerable importance in the treatment of chronic disease.

It is scarcely necessary to take any notice of the dose of this medicine, or the method of administering it. If given in solution, it ought always to be given either largely diluted, or taken after meals, or both. A very good method is to order it to be taken in a cupful of equal parts of milk and water. If, in this form, it produces anorexia, vomiting, or pain of stomach, the prescription ought to be changed; it may be combined with opium, or administered in an effervescing draught. The most common error, in prescribing the solution, is giving it in too large doses. If five drops disagree, let two or three only be given, at shorter intervals; and if there exist an imperious necessity for bringing the system under its influence, it may be given in small doses every half-hour or hour. Fortunately, the necessity for giving this powerful medicine so rapidly, does not often exist in this country; but in the East Indies, it is given in this way as an antidote against the bite of poisonous serpents. It operates in these cases as a powerful stimulant, counteracting the intensely sedative action of the animal poison; and from its permanent effects, it is much more valuable than ammonia, or any other of that class of medicines.

I will here subjoin two cases, illustrative of its action, and of the length of time it may be used.

J. A. about 40 years of age, affected with a cutaneous eruption on his hands, began the use of Fowler's solution, in doses of five drops three times a-day, to be gradually increased to ten. Being at some distance in the country, and the eruption disappearing, he continued increasing the dose to fifteen drops, instead of ten; he also neglected to call every third day, as ordered. He had continued this increased dose about a week, when he called upon me, complaining of a severe cold he supposed he had caught. I found him with a pulse of 120, particularly full, hard, and bounding; complaining of cough, attended with a feeling of oppression and tightness about the chest; there was general anasarca: and his urine was scanty and high-coloured; his whole system, in fact, seemed labouring under an excess of fluids. He was bled to ℥xx.; the blood flowed with unusual force, and was of a bright arterial colour: the bleeding was followed up by saline purgatives, and the disagreeable symptoms speedily disappeared.

The second case is illustrative of the length of time during which the arsenical oxide may be administered without inconvenience. It is a case of Lepra vulgaris, which I first saw about two years ago. The eruption covered nearly the whole body, and the scales were of unusual thickness: being complicated with an ulcer in the fauces, apparently syphilitic, and iritis of one eye, the case was at first treated with mercury and sarsaparilla. The iritis and sore throat were cured, but the eruption did not yield. He was then put on the use of the arsenical solution, in doses of five drops three times a-day; it was pushed the length of ten drops as often daily before the affection yielded. The use of the medicine was then given up: in three weeks, the eruption re-appeared; was again cured; and a second time returned. This routine was gone through many times, until my poor patient was reduced to the necessity of choosing the least of two evils, namely, the taking a daily dose of medicine. He has now been taking the solution constantly for the last year; he perceives no bad effects from its use; goes about his usual avocations; his pulse keeps steadily above a hundred, full and strong; his eye suffused and watery; palpebræ slightly inflamed; appetite good; tongue has the white chalky appearance described above; and his general aspect is that of a person in high health. He informs me, that he suffers no inconvenience from the use of the remedy, and declares his willingness to continue its use so long as it is necessary. I may here state, that though thirty drops each day were required to cure the disease, fifteen appear sufficient to keep it in check; and the latter is the dose he has been taking for some time.

On reflecting upon the above case, the question naturally occurs—How does the system eliminate the medicine? That it is expelled in some way or other is certain, and the urine being the only excretion in which we have any hope of detecting it, I intended to have made an attempt to separate it from the other constituents of that fluid. The following consideration has as yet, however, prevented me from doing so: The patient at present takes ⅛th gr. of the oxide daily; and if we assume this quantity to be equally diffused over the whole body, solids and fluids, the urine will not receive above 1-60th of it. To detect so very minute a quantity, even with all the refinement and delicacy which Christison has given the process of analysis, would, I imagine, be difficult, if not impossible."

---

